# In Vitro Evaluation of the Inhibitory Effects of Linarin on Histamine‐Induced Expression of Proinflammatory Cytokines, Mucin 5AC, and Aquaporin 5 in Human Nasal and Bronchial Epithelial Cells

**DOI:** 10.1002/kjm2.70114

**Published:** 2025-09-25

**Authors:** Xin‐Jing Mi, Jie Wang, Jian‐Qiang Qi

**Affiliations:** ^1^ Department of Pediatrics Xi'an Hospital of Traditional Chinese Medicine Xi'an China; ^2^ Department of Stomatology Dalian Rehabilitation and Convalescent Center of Joint Logistics Support Unit Dalian China; ^3^ Clinical Skills Teaching Center Fujian University of Traditional Chinese Medicine Fuzhou China

**Keywords:** allergic rhinitis, asthma, linarin, MAPK, NF‐κB

## Abstract

Allergic rhinitis and asthma are two prevalent chronic allergic conditions in children. Linarin, a glycosylated flavonoid derived from various plants, exhibits a wide range of biological activities. This study aimed to investigate the therapeutic potential of linarin against allergic rhinitis and asthma. In vitro models of allergic rhinitis and asthma were established using human nasal epithelial cells (hNECs) and human bronchial epithelial cells (BEAS‐2B), respectively. Linarin treatment inhibited histamine‐induced increases in the expression levels of p‐p65 and p‐IκBα in whole‐cell lysates, as well as p65 in nuclear lysates. The histamine‐induced activation of MAPK pathways was suppressed by linarin, as evidenced by reduced phosphorylation ratios of ERK (pERK/ERK), JNK (pJNK/JNK), and p38 (pp38/p38). ELISA results further revealed that linarin attenuated histamine‐induced secretion of proinflammatory cytokines, including IL‐6, IL‐8, and MCP‐1. Western blot and RT‐PCR analyses showed that linarin reversed histamine‐induced MUC5AC upregulation and AQP5 downregulation. Notably, the inhibitory effects of linarin were potentiated in the presence of specific inhibitors targeting NF‐κB (PDTC), ERK (U0126), p38 (SB203580), and JNK (SP600125). Collectively, these findings demonstrate that linarin suppresses histamine‐induced proinflammatory cytokine secretion, MUC5AC upregulation, and AQP5 downregulation in human nasal and bronchial epithelial cells. These effects are mediated through the inhibition of the NF‐κB and MAPK pathways. Thus, linarin may serve as a promising therapeutic agent for the treatment of allergic rhinitis and asthma.

## 1 Introduction

Allergic diseases are major concerns in childhood because they lead to many disorders such as impaired growth, sleep disorders, and reduced educational attainment. They impose a considerable health burden and negatively affect quality of life, particularly when linked with serious comorbidities [1]. Among these, allergic rhinitis and asthma are two of the most common chronic allergic disorders, frequently occurring together in children [2]. Evidence suggests that it contributes to poor asthma control, while effective treatment of rhinitis can improve asthma outcomes [3]. Therefore, developing therapies targeting both conditions is of clinical importance.

**FIGURE 1:**
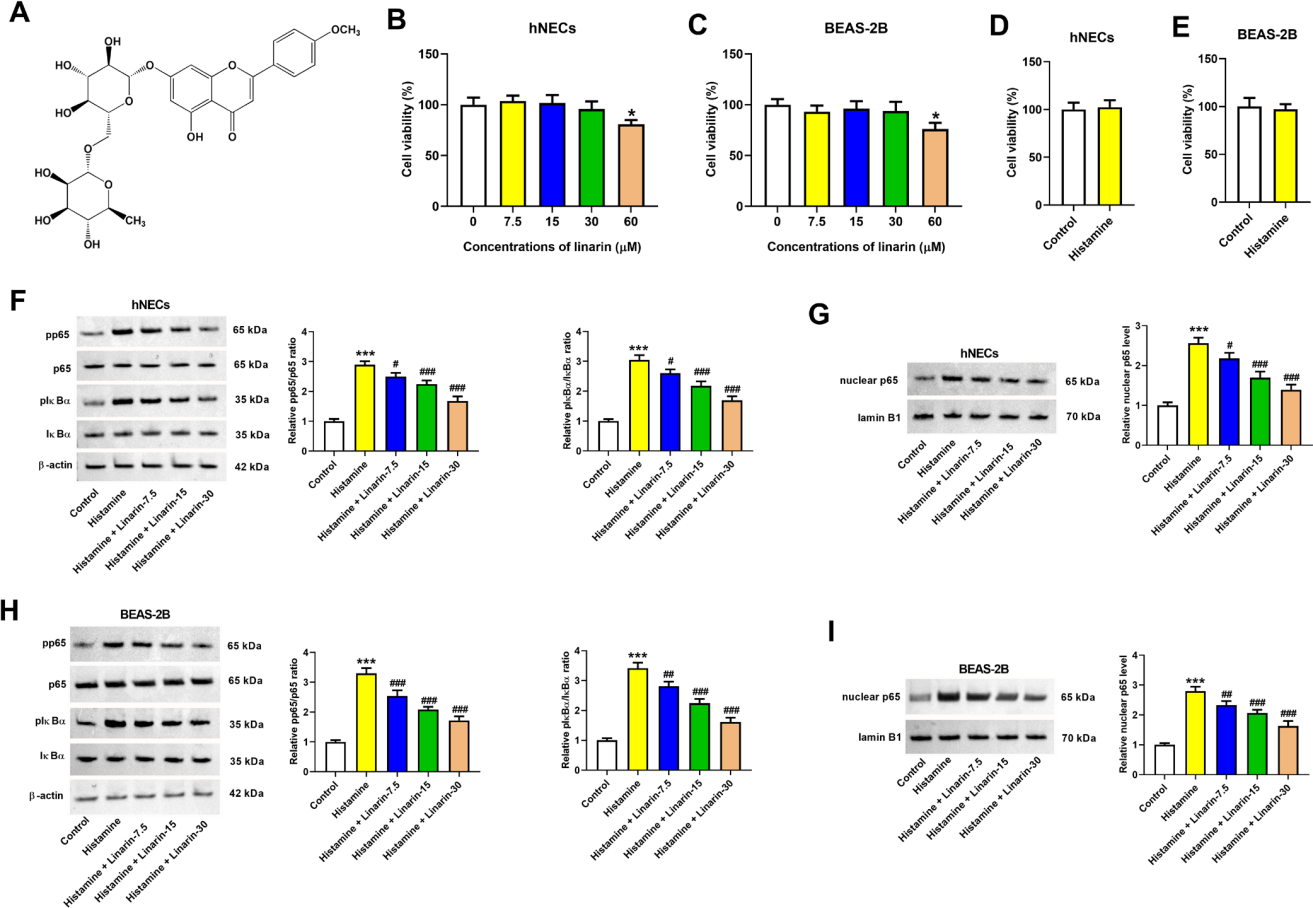
Effects of linarin on the histamine‐induced NF‐κB pathway. (A) Chemical structure of linarin. (B, C) The viability of hNECs and BEAS‐2B cells 24 h after treatment with 0, 7.5, 15, 30, or 60 μM linarin. *p < 0.05 versus control cells. (D, E) The viability of hNECs and BEAS‐2B cells after stimulation with 100 μM histamine for 24 h. (F–I) hNECs and BEAS‐2B cells were pretreated with linarin (7.5, 15, and 30 μM) for 1 h, and then stimulated with histamine (100 μM) for 24 h. The pp65, p65, pIκBα, IκBα, and nuclear p65 expression levels were measured by western blotting. ***p < 0.01 versus control group. ^#^ *p* < 0.05, ^##^ *p* < 0.01, and ^###^ *p* < 0.001 versus histamine group.

Linarin (Figure 1A) is a glycosylated flavonoid identified in plants from the *Lamiaceae* and *Asteraceae* families [4]. Phytochemical and pharmacological studies have reported various biological effects for linarin, including hepatoprotective [5], anti‐diabetic [6], neuroprotective [7], anti‐ischemic [8], osteogenic and anti‐osteoclastic [9], and anti‐tumor properties [10]. Notably, linarin has been shown to modulate inflammatory responses by inhibiting the NF‐κB and MAPK pathways [11]. Moreover, inhibition of the NF‐κB and MAPK pathways is implicated in the pathogenesis of asthma and allergic rhinitis [12, 13]. These findings suggest that linarin has therapeutic potential in treating both conditions.

Histamine, primarily stored in mast cells and basophils, plays a prominent role in the pathophysiology of allergic diseases, including asthma and allergic rhinitis [14]. Molecular features of these diseases include the upregulation of proinflammatory cytokines and MUC5AC, along with downregulation of aquaporin 5 (AQP5) [15, 16, 17]. Histamine is widely used in experimental models to mimic allergic responses due to its ability to induce these molecular changes [15, 18]. Based on this background, we speculated that linarin may inhibit histamine‐induced secretion of inflammatory cytokines and regulate the expression of MUC5AC and AQP5 by modulating the NF‐κB and MAPK pathways. This study aimed to test this hypothesis using in vitro models of allergic rhinitis and asthma.

## 2 Materials and Methods

### 2.1 Cell Culture

Human nasal epithelial cells (hNECs; Procell, Wuhan, China) and human bronchial epithelial BEAS‐2B cells (ATCC, Manassas, VA) were used in this study. hNECs and BEAS‐2B cells were cultured in DMEM containing 10% FBS (Thermo Fisher Scientific, Waltham, MA) and penicillin (10,000 units/mL)‐streptomycin (10,000 μg/mL, Thermo Fisher Scientific) at 37°C under a 5% CO_2_ atmosphere. To establish an in vitro model of allergic rhinitis and asthma, hNECs and BEAS‐2B cells were stimulated with histamine (100 μM) for 24 h. Linarin (purity ≥ 98%; ChemFaces, Wuhan, China; 7.5, 15, 30, and 60 μM) and/or inhibitors of NF‐κB (PDTC, 10 μM; Sigma‐Aldrich, St. Louis, MO, USA), ERK (U0126, 10 μM; Sigma‐Aldrich), JNK (SP600125, 10 μM; Sigma‐Aldrich), and p38 (SB203580, 30 μM; Sigma‐Aldrich) were used. The concentrations of the inhibitors were based on two previous reports [19, 20]. Dexamethasone (100 nM; Sigma‐Aldrich) was used as a positive control, and its concentration was selected based on previous studies [21, 22]. Linarin, dexamethasone, and the inhibitors were dissolved in dimethyl sulfoxide (DMSO). DMSO concentration in all experiments did not exceed 0.1% (v/v), which did not affect cell viability. The cells were pretreated with linarin and/or the inhibitors for 1 h and then stimulated with histamine (100 μM) for 24 h [20].

### 2.2 CCK‐8 Assay for Cell Viability

hNECs and BEAS‐2B cells were subjected to cell viability detection using Cell Counting Kit‐8 (Beyotime Biotechnology, Shanghai, China). Briefly, the cells were cultured overnight at a density of 1.5 × 10^3^ cells/well. Next, the cells were subjected to the CCK‐8 assay, and then the absorbance at a wavelength of 450 nm was measured using a microplate reader (Bio‐Rad Laboratories, Hercules, CA, USA). The experiments were performed three times in triplicate.

### 2.3 Western Blotting

The lysates of hNECs and BEAS‐2B cells were obtained using RIPA lysis buffer (Beyotime) containing a protease inhibitor cocktail and phosphatase (Roche). The nuclear protein extracts were prepared using a nuclear protein extraction kit (Beyotime). The proteins were then subjected to 10%–12% SDS‐PAGE to separate the indicated proteins, followed by transferring onto a PVDF membrane. Then, the western blotting was performed through incubation with specific primary antibodies against NF‐κB p65, phosphorylation of NF‐κB p65 (pp65), IκBα, phosphorylation of IκBα (pIκBα), ERK, phosphorylation of ERK (pERK), JNK, phosphorylation of JNK (pJNK), p38, phosphorylation of p38 (pp38), AQP5, CREB, phosphorylation of CREB (pCREB), or β‐actin (all agents obtained from Abcam, Cambridge, MA, USA or Cell Signaling Technology, Boston, MA, USA) at 4°C overnight. The membranes were then blotted with respective secondary antibodies (Abcam) for 1 h at room temperature. The target protein bands were finally developed with an Amersham ECL kit (Amersham, Bucks, UK). The experiments were repeated three times.

### 2.4 Immunofluorescence Staining

NF‐κB p65 distribution in hNECs and BEAS‐2B cells was detected by immunofluorescence staining. Briefly, the cells were fixed with 4% paraformaldehyde and permeabilized with 0.1% Triton X‐100. Cells were incubated with anti‐NF‐κB p65 primary antibody (1:100 dilution; Abcam) overnight, and then incubated at room temperature for 2 h with FITC‐labeled goat anti‐rabbit lgG secondary antibody (Abcam). The cell nuclei were counterstained with 4′,6‐diamidino‐2‐phenylindole (DAPI; Invitrogen, Carlsbad, CA) at room temperature for 5 min. Immunofluorescence images were captured under a fluorescence microscope (Olympus, Tokyo, Japan).

### 2.5 ELISA

After incubating with the indicated agents, the medium samples of hNECs and BEAS‐2B cells were collected and concentrated for the detection of IL‐6, IL‐8, MCP‐1, and MUC5AC. The levels of IL‐6, IL‐8, and MCP‐1 were determined using a Human IL‐6 Quantikine ELISA Kit (QK206; R&D Systems, Minneapolis, MN), Human IL‐8/CXCL8 Quantikine ELISA Kit (D8000C; R&D Systems), and Human CCL2/MCP‐1 Quantikine ELISA Kit (DCP00; R&D Systems) following the manufacturer's protocol. The MUC5AC level was determined using a Human Mucin 5 subtype AC ELISA kit (E01M0350, BlueGene, Shanghai, China) in accordance with the manufacturer's instructions. Finally, the optical density was determined using a microplate reader within 30 min. The experiments were performed three times in triplicate.

### 2.6 Real‐Time Quantitative PCR (qRT‐PCR)

Total RNA samples were extracted from hNECs and BEAS‐2B cells using TRIzol reagent (Beyotime). Then, the reverse transcription reaction was performed with SuperScript III First‐Strand Synthesis SuperMix (Thermo Fisher Scientific), as instructed by the manufacturer's protocol. qRT‐PCR was conducted using the SYBR Green PCR MasterMix (Applied Biosystems, CA, USA) on an ABI7500 FAST system. Primers used in the study included the following: MUC5AC, forward 5′‐AGTG TCCC CCAT GCAC TGA‐3′ and reverse 5′‐CAGG GGCA CAAG TTCC ACTG‐3′; AQP5, forward 5′‐AAGA AGGA GGTG TGTT CAGT TGCC TTCT TCA‐3′ and reverse 5′‐GTGT GCCG TCAG CTCG ATGG TCTT CTTC CG‐3′; GAPDH, forward 5′‐CCTC TGAC TTCA ACAG CGAC AC‐3′ and reverse 5′‐TGGT CCAG GGGT CTTA CTCC‐3′. The 2^−ΔΔCt^ method was applied to calculate the relative levels of MUC5AC and AQP5 by normalizing to an internal control GAPDH. The experiments were performed three times in triplicate.

### 2.7 Statistical Analysis

Statistical analysis of all obtained data from each experiment was performed using Prism Version 8.0.2 software (Graphpad Software, La Jolla, CA, USA). The one‐way analysis of variance (ANOVA) followed by Tukey's test was performed for the comparisons among multiple groups. Data were presented as the means ± SD. Statistical significance was defined as *p* < 0.05.

## 3 Results

### 3.1 Linarin Inhibits Histamine‐Induced NF‐κB Activation Pathway in hNECs and BEAS‐2B Cells

**FIGURE 2:**
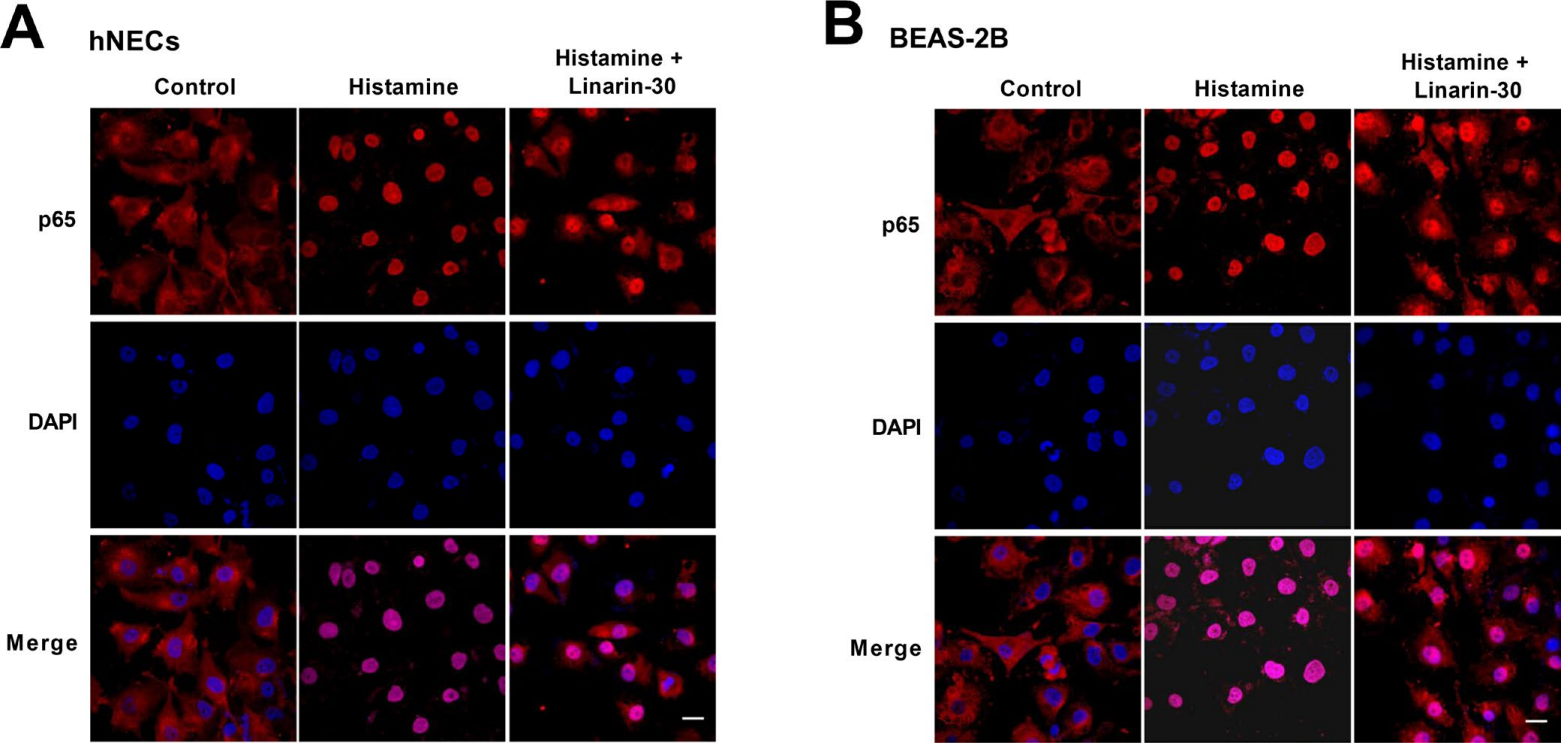
Effects of linarin on histamine‐induced nuclear translocation of NF‐κB p65. (A, B) hNECs and BEAS‐2B cells were pretreated with linarin (30 μM) for 1 h, and then stimulated with histamine (100 μM) for 24 h. The NF‐κB p65 distribution in hNECs and BEAS‐2B cells was observed by immunofluorescence. Red fluorescence identifies p65 and the cell nuclei were stained with DAPI (blue). Scale bar = 20 μm.

To assess the effect of linarin on cell viability, hNECs and BEAS‐2B cells were incubated with 0, 7.5, 15, 30, or 60 μM linarin. As shown in Figure 1B,C, cell viability was not affected by 7.5, 15, and 30 μM linarin, whereas 60 μM significantly reduced viability. Thus, 7.5, 15, and 30 μM were selected for subsequent experiments. Treatment with 100 μM histamine did not affect the viability of either hNECs or BEAS‐2B cells (Figure 1D,E). Histamine stimulation increased the pp65/p65 and pIκBα/IκBα ratios and nuclear p65 expression levels in hNECs, which were attenuated by linarin in a dose‐dependent manner (Figure 1F,G). Similar inhibitory effects were observed in BEAS‐2B cells (Figure 1H,I). Immunofluorescence analysis showed that p65 was primarily localized in the cytoplasm under basal conditions but translocated to the nucleus following histamine treatment. Linarin treatment prevented this translocation in both cell types (Figure 2A,B). These findings indicate that linarin suppresses histamine‐induced activation of the NF‐κB pathway in hNECs and BEAS‐2B cells.

### 3.2 Linarin Suppresses Histamine‐Induced MAPK Pathway Activation in hNECs and BEAS‐2B Cells

**FIGURE 3:**
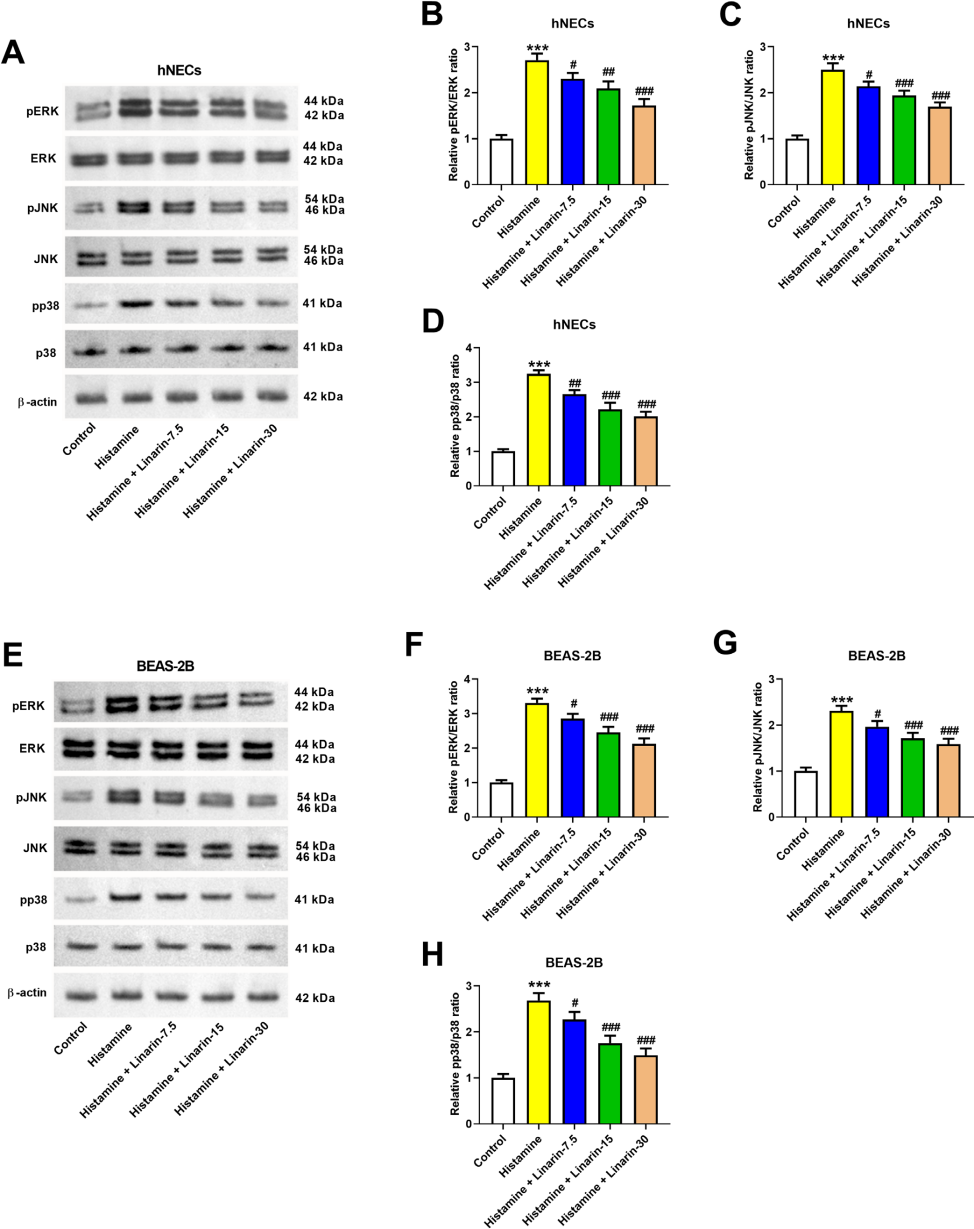
Effects of linarin on histamine‐induced MAPK pathways. hNECs and BEAS‐2B cells were pretreated with linarin (7.5, 15, and 30 μM) for 1 h, and then stimulated with histamine (100 μM) for 24 h. The effects of linarin on histamine‐induced MAPK pathways were assessed by detecting the expression levels of pERK, ERK, pJNK, JNK, pp38, and p38 in hNECs (A–D) and BEAS‐2B (E–H) cells by western blotting. ***p < 0.01 versus control group. ^#^ *p* < 0.05, ^##^ *p* < 0.01, and ^###^ *p* < 0.001 versus histamine group.

We next examined whether linarin modulates histamine‐induced MAPK pathway activation. Histamine increased phosphorylation levels of ERK, JNK, and p38, as indicated by elevated pERK/ERK, pJNK/JNK, and pp38/p38 ratios. Pretreatment with linarin (7.5, 15, or 30 μM) reversed these changes in hNECs (Figure 3A–D). Similar inhibitory effects were observed in histamine‐stimulated BEAS‐2B cells (Figure 3E–H).

### 3.3 Linarin Inhibits Histamine‐Induced Proinflammatory Cytokine Secretion by Repression of the NF‐κB and MAPK Pathways

**FIGURE 4:**
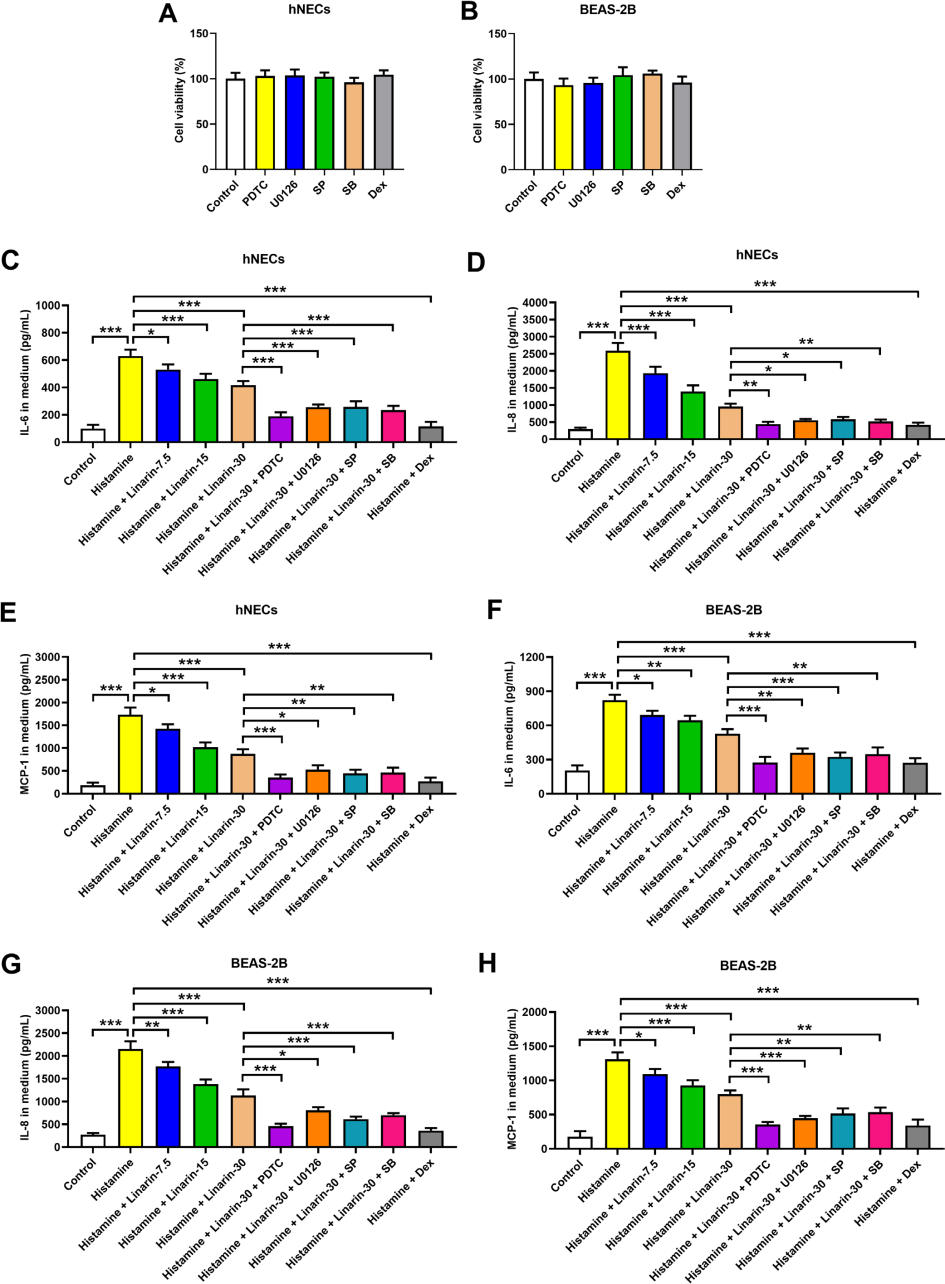
Roles of NF‐κB and MAPK pathways in regulating inflammatory cytokine secretion in linarin‐treated cells. (A, B) hNECs and BEAS‐2B cells were treated with dexamethasone (100 nM) or the inhibitors of NF‐κB (PDTC, 10 μM), ERK (U0126, 10 μM), JNK (SP600125, 10 μM), and p38 (SB203580, 30 μM) for 24 h. The cytotoxicity effects of dexamethasone and these inhibitors were evaluated by CCK‐8 assay. (C–H) hNECs and BEAS‐2B cells were pretreated with linarin or/and the inhibitors of NF‐κB (PDTC, 10 μM), ERK (U0126, 10 μM), JNK (SP600125, 10 μM), and p38 (SB203580, 30 μM) for 1 h, and then stimulated with histamine (100 μM) for 24 h. The secretion levels of IL‐6, IL‐8, and MCP‐1 in the medium were detected by ELISA. Cells pretreated with dexamethasone (100 nM) for 1 h and then stimulated with histamine (100 μM) for 24 h were used as a positive control. *p < 0.05, **p < 0.01, and ***p < 0.001 versus relative group. SP, SP600125. SB, SB203580. Dex, dexamethasone.

To validate the involvement of the NF‐κB and MAPK pathways in linarin's anti‐inflammatory effects, cells were pretreated with specific inhibitors targeting NF‐κB (PDTC, 10 μM), ERK (U0126, 10 μM), JNK (SP600125, 10 μM), and p38 (SB203580, 30 μM) to block the activation of relative pathways. Cells were also pretreated with dexamethasone (100 nM) as a positive control. None of these agents showed cytotoxic effects (Figure 4A,B). Histamine stimulation increased IL‐6, IL‐8, and MCP‐1 secretion in hNECs, which was suppressed by linarin in a dose‐dependent manner. The inhibitory effects of linarin were further enhanced by each of the pathway inhibitors (Figure 4C–E). Similar patterns were observed in BEAS‐2B cells (Figure 4F–H). As a positive control, dexamethasone suppressed histamine‐induced secretion of IL‐6, IL‐8, and MCP‐1 in hNECs and BEAS‐2B cells (Figure 4C–H). These results suggest that linarin inhibits histamine‐induced cytokine secretion by targeting the NF‐κB and MAPK pathways.

### 3.4 Linarin Attenuates Histamine‐Induced MUC5AC Upregulation by Repression of the NF‐κB and MAPK Pathways

**FIGURE 5:**
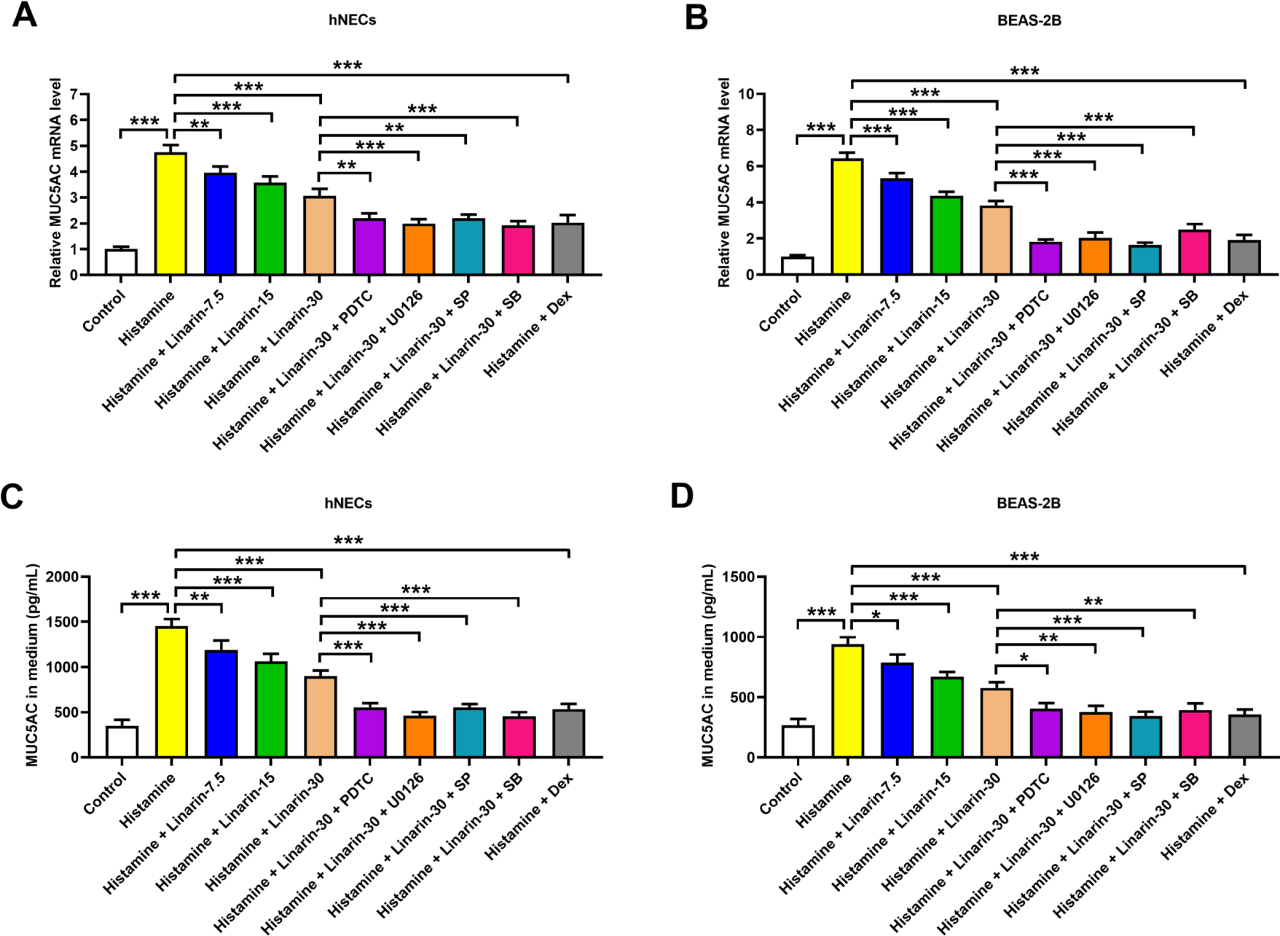
Roles of NF‐κB and MAPK pathways in regulating the MUC5AC expression in linarin‐treated cells. hNECs and BEAS‐2B cells were pretreated with linarin or/and the inhibitors of NF‐κB (PDTC, 10 μM), ERK (U0126, 10 μM), JNK (SP600125, 10 μM), and p38 (SB203580, 30 μM) for 1 h, and then stimulated with histamine (100 μM) for 24 h. Cells pretreated with dexamethasone (100 nM) for 1 h, and then stimulated with histamine (100 μM) for 24 h were used as a positive control. (A and B) RT‐PCR was performed to detect the mRNA levels of MUC5AC in both hNECs and BEAS‐2B cells. (C and D) ELISA was conducted to measure the secretion levels of MUC5AC in a cell culture medium of hNECs and BEAS‐2B cells. *p < 0.05, **p < 0.01, ***p < 0.001 versus relative group. SP, SP600125. SB, SB203580.

Histamine treatment induces the expression of MUC5AC, a key mucin component of airway mucus, in nasal and lung tissues, potentially contributing to the progression of allergic respiratory diseases [23, 24]. Consistent with previous reports, histamine significantly upregulated MUC5AC mRNA expression in hNECs and BEAS‐2B cells. However, pretreatment with linarin suppressed this upregulation, and the inhibitory effect was enhanced by blocking the NF‐κB and MAPK pathways (Figure 5A,B). ELISA confirmed the reduced MUC5AC secretion in linarin‐treated cells, further potentiated by the inhibitors of the NF‐κB and MAPK pathways (Figure 5C,D). As a positive control, dexamethasone also suppressed histamine‐induced MUC5AC expression and secretion in hNECs and BEAS‐2B cells (Figure 5A–D).

### 3.5 Linarin Reverses Histamine‐Induced AQP5 Downregulation by Repression of the NF‐κB and MAPK Pathways

**FIGURE 6:**
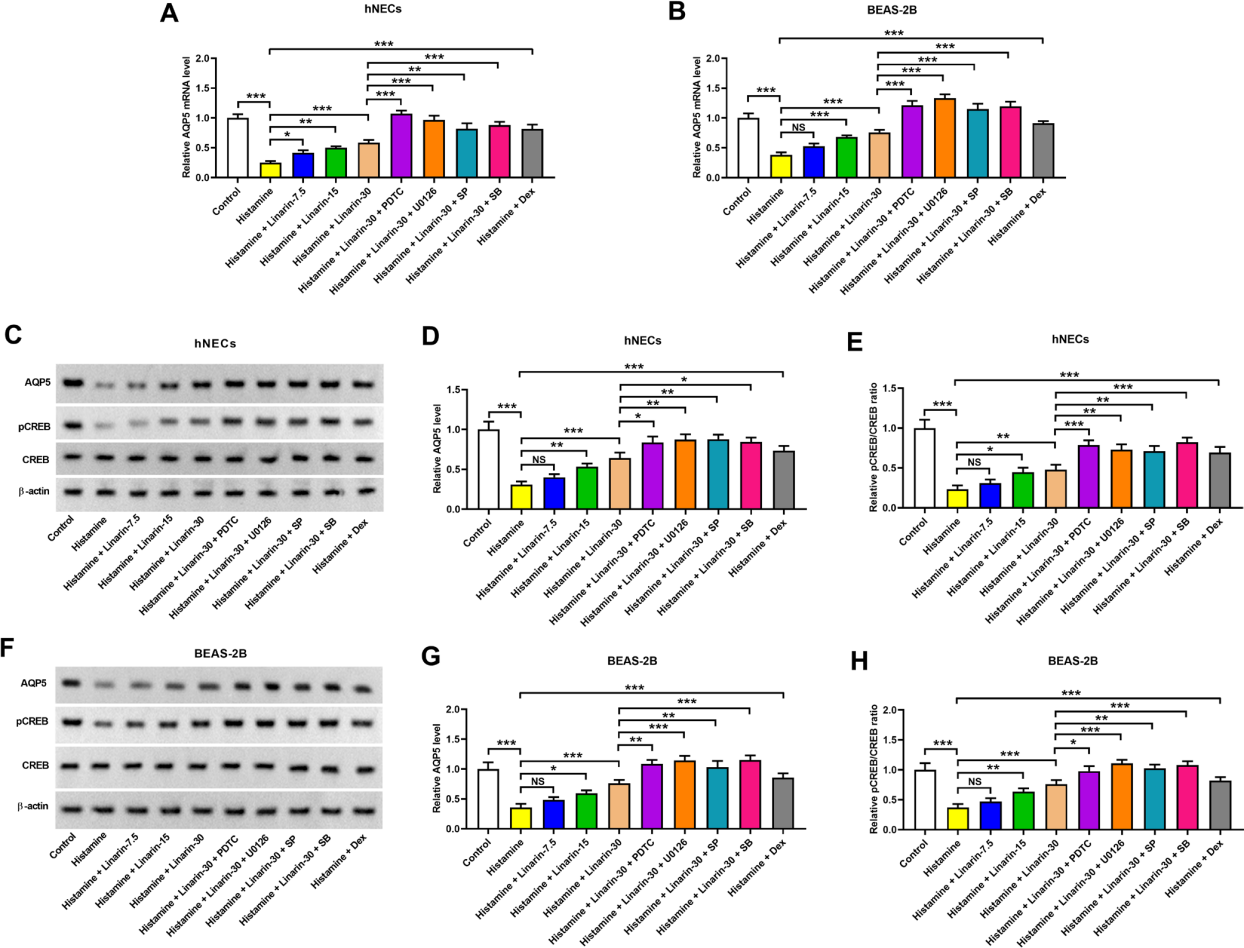
Roles of the NF‐κB and MAPK pathways in regulating the AQP5 expression in linarin‐treated cells. hNECs and BEAS‐2B cells were pretreated with linarin or/and the inhibitors of NF‐κB (PDTC, 10 μM), ERK (U0126, 10 μM), JNK (SP600125, 10 μM), and p38 (SB203580, 30 μM) for 1 h and then stimulated with histamine (100 μM) for 24 h. Cells pretreated with dexamethasone (100 nM) for 1 h, and then stimulated with histamine (100 μM) for 24 h were used as a positive control. (A and B) RT‐PCR was performed to detect the mRNA levels of AQP5 in both hNECs and BEAS‐2B cells. (C–H) Western blotting was conducted to measure the expression levels of AQP5, CREB, and pCREB in hNECs and BEAS‐2B cells. *p < 0.05, **p < 0.01, ***p < 0.001 versus relative group. “NS,” no significance. SP, SP600125. SB, SB203580.

Histamine has been shown to downregulate AQP5 expression in hNECs and BEAS‐2B cells by inhibiting CREB phosphorylation at serine 133 [18, 25]. Our results confirmed histamine‐induced AQP5 downregulation in both hNECs and BEAS‐2B cells. Linarin restored AQP5 mRNA expression level, and this effect was amplified by inhibitors of the NF‐κB and MAPK pathways (Figure 6A,B). Western blotting also proved that histamine reduced both AQP5 and phosphorylated CREB (p‐CREB) protein levels in hNECs and BEAS‐2B cells. Linarin pretreatment restored their expression, and co‐treatment with NF‐κB and MAPK pathway inhibitors further enhanced this restoration (Figure 6C–H). As a positive control, dexamethasone attenuated histamine‐induced decrease in the expression levels of AQP5 mRNA, AQP5 protein, and p‐CREB in hNECs and BEAS‐2B cells (Figure 6A–H).

## 4 Discussion

Allergic diseases such as allergic rhinitis and allergic asthma result from a complicated interplay among multiple inflammatory cells, including basophils, mast cells, lymphocytes, dendritic cells, and neutrophils in response to diverse environmental or allergic stimuli [26]. These cells generate various inflammatory mediators, including histamine, chemokines, eicosanoids, cytokines, and reactive oxygen species [27]. Among these mediators, mast cell histamine plays a central role in promoting the development of allergic‐related inflammatory diseases [27]. When histamine is released, it can bind to the receptors on the surface of epithelial cells, triggering a series of intracellular signaling cascades and thus activating those epithelial cells [28]. Histamine can also stimulate epithelial cells to secrete cytokines, thereby promoting cell activation and inflammatory responses [29]. In this study, we found that linarin suppresses histamine‐induced proinflammatory cytokine secretion, MUC5AC upregulation, and AQP5 downregulation in human nasal and bronchial epithelial cells. Allergic rhinitis and asthma share multiple common pathogenetic and pathophysiological mechanisms and frequently coexist [30]. Therefore, we believe that our findings have provided valuable information for the discovery of a drug that can treat both allergic rhinitis and asthma.

The NF‐κB and MAPK pathways are widely recognized to be involved in the development of allergic rhinitis and asthma [12, 13]. During the past decades, numerous active ingredients isolated from Traditional Chinese Medicine have shown efficacy against allergic disorders (allergic rhinitis and asthma) by modulating inflammatory pathways. Tussilagone, a sesquiterpene compound separated from Farfarae Flos, suppresses allergic responses in ovalbumin (OVA)‐induced allergic rhinitis in guinea pigs by inhibiting allergic and inflammatory‐related pathways in mast cells, including the NF‐κB, ERK, and p38 MAPK pathways [31]. Paeoniflorin, an active ingredient purified from *Paeonia lactiflora* Pall roots, inhibits mast cell‐mediated allergic inflammation by suppressing the NF‐κB and MAPK signaling pathways [32]. Leonurine, a natural alkaloid extracted from *Herba leonuri*, exerts an anti‐asthma effect in lipopolysaccharide (LPS)‐induced RAW264.7 cells and an OVA‐induced asthmatic mouse model by regulating the p38 MAPK/NF‐κB signaling pathway [33]. In both in vitro and in vivo assays, Ginsenoside Rh1 ameliorates OVA/LPS‐induced asthma and allergic inflammation by blocking the activation of MAPK and NF‐κB signaling pathways [12]. In line with these studies, our results indicate that linarin inhibited histamine‐induced activation of the NF‐κB and MAPK pathways in hNECs and BEAS‐2B cells.

The polymeric mucin MUC5AC is one of the integral components of airway mucus secreted by airway epithelia that has been found to be elevated in allergic rhinitis and asthma [34, 35]. Alteration in MUC5AC levels contributes to mucus dysfunction in allergic rhinitis or asthma [36, 37]. Aquaporins (AQPs) are a family of transmembrane channel proteins closely involved in maintaining the fluid balance of the airway. AQP5, an important member of the AQPs family, is a key molecule related to the secretion of serous glands. It is evident that AQP5 plays a critical role in regulating fluid secretion during allergic inflammation [15]. In addition to the increased secretion of proinflammatory cytokines, histamine induces MUC5AC upregulation and AQP5 downregulation during the early phase of allergic respiratory diseases [15, 21]. Previous studies have found that AQP5 expression and distribution, and MUC5AC expression during allergic inflammation are regulated by multiple signaling pathways, including NF‐κB and MAPK pathways [38, 39, 40]. Our results unveiled that linarin suppressed histamine‐induced proinflammatory cytokines, MUC5AC upregulation, and AQP5 downregulation, which were enhanced by the inhibitors of NF‐κB and MAPK pathways. These roles of linarin, together with the aforementioned inhibitory effects on the NF‐κB and MAPK pathways, suggest that these pathways are involved in the effects of linarin. Despite these promising findings, the current study has several limitations. First, the effects of linarin have not been validated in animal models of allergic rhinitis and asthma. Future in vivo studies are needed to establish its therapeutic potential. Second, the precise molecular mechanisms by which linarin regulates NF‐κB and MAPK pathways remain unclear, and this should be clarified in the future. Third, additional pathways beyond NF‐κB and MAPK pathways are involved in allergic rhinitis and asthma. Whether these other molecular mechanisms contribute to the anti‐allergic effects of linarin needs to be determined.

**FIGURE 7:**
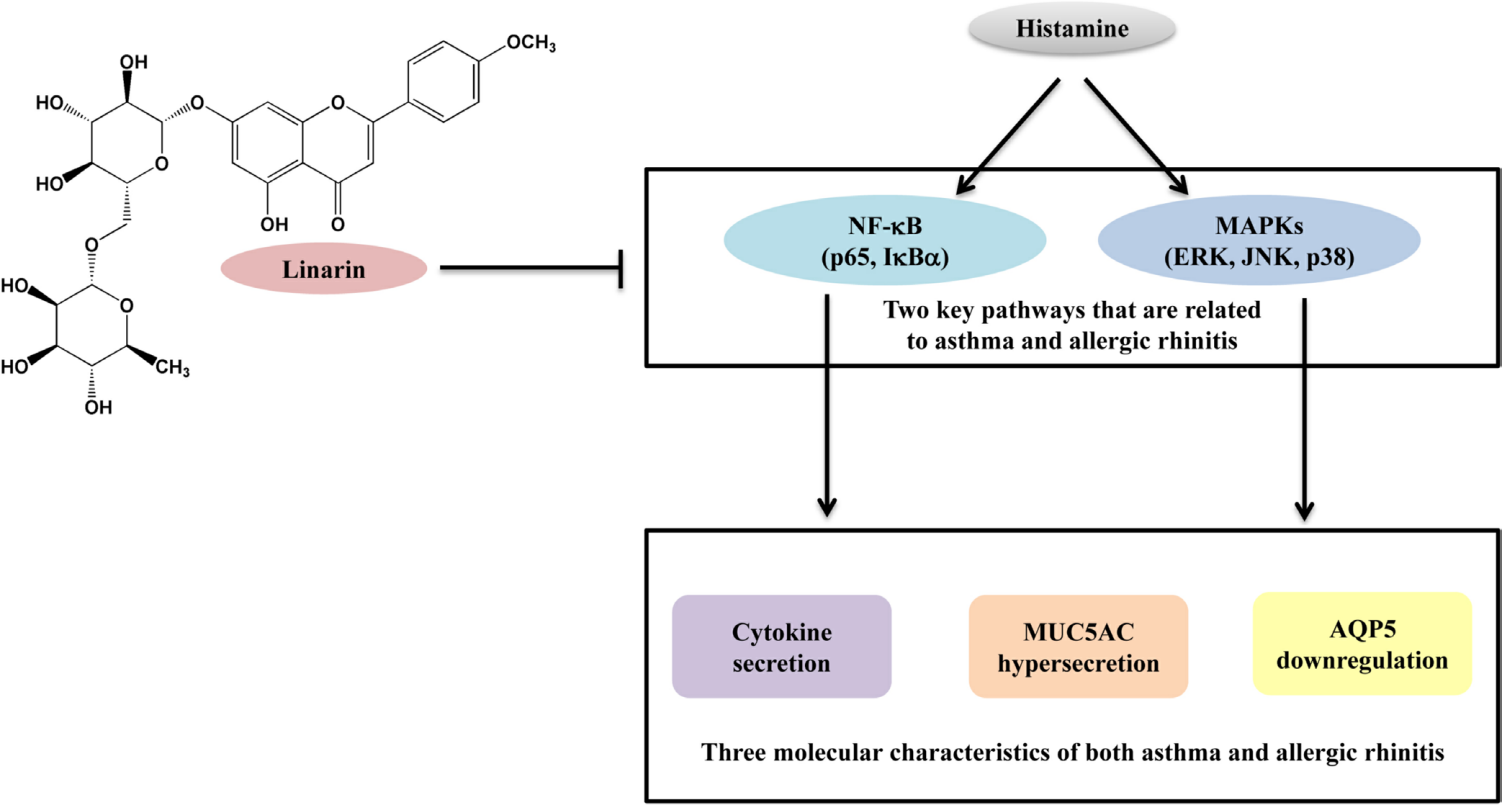
Mechanism underlying the effects of linarin on human airway epithelial cells in response to histamine stimulation. Linarin inhibited histamine‐induced activation of NF‐κB and MAPK pathways in human nasal and bronchial epithelial cells, which contributed to its effects on cytokine secretion, MUC5AC upregulation, and AQP5 downregulation.

In summary, linarin inhibited histamine‐induced proinflammatory cytokine secretion, MUC5AC upregulation, and AQP5 downregulation in human nasal and bronchial epithelial cells by inhibiting the NF‐κB and MAPK pathways (Figure 7). These findings suggest that linarin may serve as a promising therapeutic agent for ameliorating allergic rhinitis and asthma.

## Conflicts of Interest

The authors declare no conflicts of interest.

## Data Availability

The data that support the findings of this study are available from the corresponding author upon reasonable request.
